# Load and distinctness interact in working memory for lexical manual gestures

**DOI:** 10.3389/fpsyg.2015.01147

**Published:** 2015-08-13

**Authors:** Mary Rudner, Elena Toscano, Emil Holmer

**Affiliations:** Linnaeus Centre HEAD, Swedish Institute for Disability Research, Department of Behavioural Sciences and Learning, Linköping University, Sweden

**Keywords:** working memory, manual gestures, load, distinctness, resolution, computer games

## Abstract

The Ease of Language Understanding model (Rönnberg et al., 2013) predicts that decreasing the distinctness of language stimuli increases working memory load; in the speech domain this notion is supported by empirical evidence. Our aim was to determine whether such an over-additive interaction can be generalized to sign processing in sign-naïve individuals and whether it is modulated by experience of computer gaming. Twenty young adults with no knowledge of sign language performed an *n*-back working memory task based on manual gestures lexicalized in sign language; the visual resolution of the signs and working memory load were manipulated. Performance was poorer when load was high and resolution was low. These two effects interacted over-additively, demonstrating that reducing the resolution of signed stimuli increases working memory load when there is no pre-existing semantic representation. This suggests that load and distinctness are handled by a shared amodal mechanism which can be revealed empirically when stimuli are degraded and load is high, even without pre-existing semantic representation. There was some evidence that the mechanism is influenced by computer gaming experience. Future work should explore how the shared mechanism is influenced by pre-existing semantic representation and sensory factors together with computer gaming experience.

## Introduction

Working memory is the ability to keep information in mind for a limited period of time while processing it (Baddeley, 2012). There is a close connection between working memory and communication which builds on the need to maintain and process information during receptive and productive language processing (Majerus, 2013) and in many ways, the functionality of working memory seems to be adapted to communication needs (Baddeley et al., 1998). Working memory for speech based language has been studied extensively and it is known that capacity is greater for words than non-words (Hulme et al., 1991) and influenced by the phonological structure of to-be-remembered items (Baddeley, 2012). There is some evidence that these effects generalize to sign language but the overall picture is not clear cut (Rudner et al., under review). Beyond linguistic aspects, working memory is influenced by memory load, operationalized either as how many, or how long, items need to be maintained, as well as the distinctness of the presented items, or how difficult it is to perceive them (Barch et al., 1997). Further, computerized training can modulate the effect of increased working memory load (Dahlin et al., 2008) and videogaming can improve cognition (Bavelier and Davidson, 2013). The purpose of the present study is to further our understanding of the language modality specificity of working memory by investigating the interplay of load and distinctness in working memory for manual gestures and its association with experience of computer games. Here, load is operationalized as the number of items maintained and distinctness as visual resolution on presentation.

Everyday listening situations are often noisy which means that the quality or distinctness of the language signal may be reduced (Mattys et al., 2012). Listening to speech in noise is more cognitively demanding, than listening to speech in quiet, especially for individuals with hearing loss (Rudner and Lunner, 2014), and generates greater neural activation throughout language processing regions (Scott and McGettigan, 2013). Individuals with greater working memory capacity are better at understanding speech in noise (Rudner et al., 2011; Zekveld et al., 2011) and show less activation in language processing regions, suggesting that the neural mechanisms supporting speech understanding in noise are more efficient in this group (Zekveld et al., 2012). This set of findings supports the notion expressed in the Ease of Language Understanding model (ELU; Rönnberg et al., 2013) that mismatch arises when the incoming language signal cannot be rapidly and automatically associated with the contents of long-term memory, and that limited working memory resources are engaged in deciphering the message. According to the ELU model (Rönnberg et al., 2013), mismatch increases working memory load because degraded, and thus indistinct, elements of the speech signal need to be held longer in working memory before they can be disambiguated by accessing the corresponding item in the mental lexicon. In other words, an indistinct speech signal actually causes greater working memory load by increasing the length of time individual items need to be maintained in working memory before speech understanding is achieved, reducing the portion of total resources available for processing new items entering the system and making them more vulnerable to mismatch. Thus, the ELU model specifically predicts an over-additive (and not under-additive) interaction between load and distinctness. The intertwining of distinctness and load during speech understanding in noise paradigms makes it hard to distinguish the underlying mechanisms.

In a set of studies from our lab (Mishra et al., 2013a,b, 2014), participants were presented with auditory 13-item lists of two-digit numbers and required to strategically select and report back two of those numbers when the list ended. Load was manipulated by requiring the participants to additionally report in half of the trials the dummy number which was always the first item in the list. Distinctness was manipulated by presenting the items with and without background noise. Because we were interested in the effects of low-level noise on cognition, the signal to noise ratio (SNR) was adapted so that items were audible. In other words, distinctness was still relatively high. Both manipulations reduced performance, but there was no interaction between load and distinctness and thus no evidence of a shared mechanism.

In a functional magnetic resonance imaging (fMRI) study, Barch et al. (1997) also investigated the interaction of distinctness and load using a task in which a sequence of letters is presented and the participant is instructed to respond to a target letter but only when it is preceded by a particular cue letter. This task loads on working memory by requiring the participant to keep the cue letter in mind until the target letter has been presented. Load was manipulated by adapting the retention interval between cue and target, and distinctness was manipulated by removing pixels from the target. Results showed that increasing load while keeping distinctness constant was associated with greater activation of the dorsolateral prefrontal cortex. On the other hand, decreasing distinctness while keeping load constant was associated with greater activation of the anterior cingulate. Thus, the results of the study by Barch et al. (1997) suggested that the neural mechanisms underpinning load and distinctness in working memory are separate. However, it is possible that the load and distinctness manipulations in the study by Barch et al. (1997) were not strong enough to trigger a shared mechanism.

In a more recent magnetoencephalography (MEG) study, Obleser et al. (2012) investigated the combined effects of distinctness and load on the neural mechanisms underpinning working memory for digits by studying neural oscillations. In particular, changes in power in low frequency oscillations in the alpha band were used as an index of working memory load. Distinctness was manipulated using noise-vocoding at 4, 8, and 16 bands. At four bands, speech is hard to understand but digits can still be identified because they belong to a small closed set. Load was manipulated by requiring the participants to retain two, four, or six items in working memory. A significant interaction was found between load and distinctness, revealing that when load was high and distinctness low there was an increase in alpha power in temporo-parietal regions. This interaction provides evidence of a shared mechanism. Taken together, evidence suggests that although load and distinctness appear to be supported by separate mechanisms there is a threshold at which a joint mechanism may be revealed empirically.

Working memory processing is supported by a load-sensitive neural network including the dorsolateral prefrontal regions identified as load-sensitive by Barch et al. (1997) and parietal regions (Ma et al., 2014) adjacent to that supporting the interaction of load and distinctness (Obleser et al., 2012). This applies across the language modalities of sign and speech (Rudner et al., 2009). Signed languages are natural languages in the visuospatial domain with vocabulary and grammatical structure that differ from those of the surrounding spoken languages (Emmorey, 2002). Working memory for sign language additionally elicits modality-specific neural activation in the parietal lobes bilaterally (Rönnberg et al., 2004; Buchsbaum et al., 2005; Rudner et al., 2007; Bavelier et al., 2008; Pa et al., 2008), possibly reflecting activation of a capacity-limited store for representation of the visual scene (Todd and Marois, 2004; Rudner, 2015). Lexicality influences the neurocognitive processing of manual gestures, even in individuals with no knowledge of sign language (Cardin et al., 2015). Further, knowledge of a signed language enhances working memory for the signs of that language, demonstrating that pre-existing lexical representation influences working memory processing of lexical signs (Rudner et al., under review). Moreover, although increasing load reduces the capacity of working memory for signs, this effect is smaller for deaf signers than for hearing signers or non-signers (Rudner et al., under review). This means that sign language allows us not only to study whether the shared mechanism supporting load and distinctness during speech processing generalizes across language modalities, but also whether it is dependent on pre-existing lexical representation. According to flexible resource models (Ma et al., 2014), the quality of input, e.g., distinctness, influences working memory processing, even when semantic representations are absent. This suggests that the over-additive interaction between load and distinctness predicted by the ELU model (Rönnberg et al., 2013) should not only generalize to sign language but may also be observable even when pre-existing representation is lacking. Thus, in order to isolate the interaction of load and distinctness in working memory for manual gestures in the present study, we presented to non-signers to-be-remembered items that were lexical signs. This allowed us to control for any effects of lexicality and pre-existing semantic representation and their potential interactions.

It is established practice that sign language interpreters choose dark clothes to contrast with their signing hands, and ensure good lighting and an unobstructed line of sight to those requiring signed translation. This suggests that poor contrast and masking are sources of visual noise that impact on visual communication. Although neither deafness nor sign language use seem to be associated with changes in contrast sensitivity (Finney and Dobkins, 2001), the data compression applied during digital video communication, used frequently by signers, may influence the quality of communication (Agrafiotis et al., 2003). However, little empirical work has addressed these issues. An early study (Pavel et al., 1987) found that adding digitally generated Gaussian noise to videos of individual lexical signs reduced the ability of deaf sign language users to identify them. In particular, a critical point was observed at root mean square SNR of 0.5.

Working memory for sign language has been shown to display some of the characteristics of working memory for speech based language (Rudner et al., 2009, 2010; Andin et al., 2013), including an effect of load (Rudner et al., under review). However, it has not hitherto been investigated whether the effect of load interacts with the distinctness of manual gestures. The ELU model is a multimodal model of working memory; in other words, it predicts similar phenomena across the language modalities of sign and speech (Rönnberg, 2003; Rönnberg et al., 2013). Based on empirical findings relating to the role of working memory during speech understanding under adverse conditions, this model predicts an over-additive interaction between working memory load and reduced distinctness. As we have argued, in the speech domain this is because an indistinct input signal causes greater working memory load by increasing the length of time individual items need to be maintained in working memory before speech understanding is achieved, reducing the portion of total resources available for processing new items entering the system and making them more vulnerable to mismatch. In the case of manual gestures, with no pre-existing representation in semantic long-term memory, mismatch will prevail. According to flexible resource models (Ma et al., 2014), the quality of input, e.g., distinctness, is important not only for achieving match but also for working memory processing as such. Thus, we predict that decreasing the distinctness of manual gestures will increase working memory load, resulting in an over-additive interaction between these two factors, even for non-signers with no corresponding representations. The main aim of the present study is to test this prediction.

Dahlin et al. (2008) showed that computerized training of updating skills led to better working memory performance when load was high. However, cognitive training programs are time-consuming and complicated to administer, and improvements in trained skills seldom transfer to untrained skills (Owen et al., 2010). Meanwhile, videogaming has become a major pastime and there is increasing evidence that playing videogames is associated with robustly enhanced visuospatial (Bavelier and Davidson, 2013) and executive (Anguera et al., 2013) skills. Thus, in the present study we asked the participants to report their experience of playing computer games and investigated whether this was associated with performance on the working memory task based on manual gestures.

As in a number of recent studies from our lab (Rudner, 2015; Rudner et al., under review), we opted to use an *n*-back working memory paradigm (Cohen et al., 1994) to investigate effects of load and distinctness on working memory performance. In the *n*-back task, series of items are presented and the task of the participant is to determine whether the current item matches the items presented n steps back in the series and make a “yes” or “no” button-press response. For example, if *n* = 1, the current item is compared to the immediately preceding item, if *n* = 2, the current item is compared to the last item but one. This task makes the temporary maintenance and processing demands that characterize working memory (Ma et al., 2014) and working memory load is determined by n; the greater the magnitude of n, the greater the working memory load. The stimulus items were videorecordings of lexical signs that were presented either at full or with reduced resolution to manipulate distinctness.

We predicted that reducing the resolution of the sign stimuli would reduce performance on the *n*-back task. Further, we predicted that increasing memory load by increasing n would reduce performance. Moreover, we predicted an over-additive interaction such that the effect of reduced resolution would be greater when working memory load was high, empirically revealing the shared mechanism, proposed by the ELU model (Rönnberg et al., 2013) and showing that it is not dependent on pre-existing semantic representation. Finally, we predicted that experience of playing computer games would be associated with working memory for manual gestures, especially when load was high and resolution low.

## Materials and Methods

### Participants

Twenty hearing participants (10 females) between 19 and 25 years (*M* = 22, SD = 1.8) took part in the study. They had no knowledge of any sign language and reported no hearing impairment. They had normal or corrected to normal vision and performed within the normal range on the Block Design subtest of WAIS-IV (Wechsler, 2008). They were all international students from Europe (18) and Asia (two), fluent in English, at Linköping University, Sweden. The study was conducted in accordance with the provisions of the Swedish Act (2003:460) concerning the Ethical Review of Research Involving Humans. Informed consent was given by all participants.

### Materials

**FIGURE 1:**
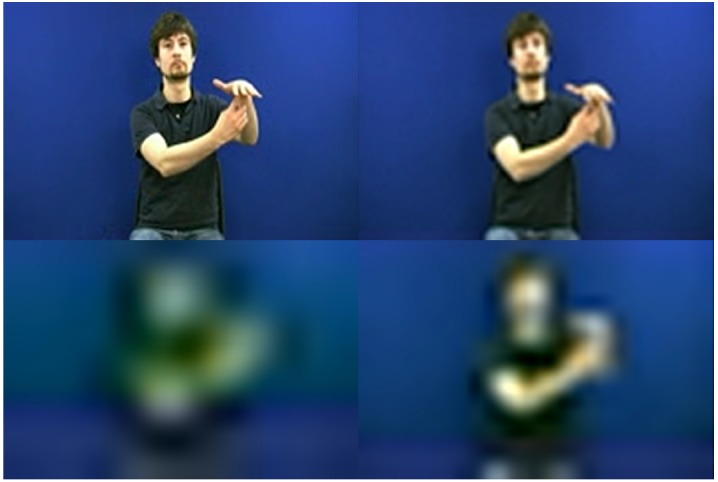
**Example of one lexical sign at resolutions R2–R5 with decreasing resolution clockwise from upper left.** R2 (180 × 120 pixels); R3 (90 × 60 pixels); R4 (24 × 16 pixels); R5 (12 × 8 pixels).

The stimulus material consisted of 90 video-recorded manual gestures, each with a duration of 2–3 s. Forty-five of the gestures constituted signs lexicalized in British Sign Language and the other 45 were signs lexicalized in Swedish Sign Language. They were all generated by a male, deaf native signer of German Sign Language who was unfamiliar with both languages. Thus, the stimuli were all natural signs and did not differ in the degree to which they were produced with a foreign accent. The materials were developed in connection with a larger project (see Cardin et al., 2013). The distinction between languages is unimportant for the purposes of the present study and the British Sign Language and Swedish Sign Language materials are balanced across stimulus lists. Each of the stimuli was processed to adapt the resolution. There were five different levels of resolution: R1 (720 × 480 pixels); R2 (180 × 120 pixels); R3 (90 × 60 pixels); R4 (24 × 16 pixels); R5 (12 × 8 pixels), see Figure 1.

Ten lists of 45 stimuli each were assembled, five for each of the two load levels of the *n*-back working memory task. Each list was available with each of the five different levels of resolution. Levels of resolution were held constant within lists. All stimuli were presented at the center of a computer screen with a constant video resolution of 1280 × 800 pixels, irrespective of the resolution of the individual stimuli.

### Experimental Task and Design

An *n*-back task was used in the present study (Cohen et al., 1994). N was either one (low load) or two (high load). During the *n*-back task, lists of videos were presented with a time between stimulus onsets of 4 s and the participant was instructed to determine for each video whether it was identical to the previous video (1-back) or the previous video but one (2-back). They pressed one key for a positive response and another key for a negative response. The dependent measure was *d*′ (Stanislaw and Todorov, 1999). No feedback was given.

The within subjects experimental design was 2 *n*-back (1-back, 2-back) × 5 resolution (R1–R5). Each participant performed each of the two *n*-back tasks five times, once with each level of resolution, and each time with a different list. *N*-back was blocked so that 10 of the participants performed the 1-back task followed by the 2-back task while order was reversed for the other 10. The assignment of lists to resolutions was balanced and the order of resolutions within blocks was pseudorandomized.

### Procedure

When the participants arrived at the laboratory, they were informed about the study and gave their written consent to participation. After providing demographic information, including how many hours a day they spent playing computer games, they performed a set of tests reported elsewhere. The test of working memory for manual gestures (*n*-back experiment) was performed at a second test session 1 month later. The *n*-back experiment was run using DMDX software (version 4.3.0.1, Forster and Forster, 2003) and took approximately 15 min to complete. The participants performed one training list for the relevant task before each block.

## Results

### *N*-Back Experiment

**FIGURE 2:**
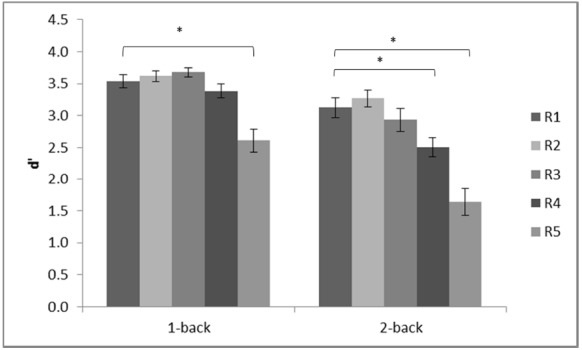
**Mean ***d***′ in each of the conditions of the n-back experiment.** R1 (720 × 480 pixels); R2 (180 × 120 pixels); R3 (90 × 60 pixels); R4 (24 × 16 pixels); R5 (12 × 8 pixels). Error bars show standard error. Brackets show significant differences, *p < 0.05.

Inspection of the *d*′ scores revealed that the scores of one of the participants in the low load condition (1-back) were more than two standard deviations below the mean across all five conditions. The participant performed the 2-back task first without any difficulty (all scores were within the same range as those of the other participants) but confirmed that she was tired and did not pay attention to the subsequent 1-back task. It was therefore decided to replace the 1-back scores of that participant with group mean for the analyses. The adjusted *d*′ scores are shown in Figure 2.

A repeated measures analysis of variance (ANOVA) was computed on *d*′ scores with two within subject factors: working memory load at two levels (low, high) and resolution at five levels (R1–R5). The ANOVA revealed a significant main effect of working memory load, *F*(1,19) = 33.63, MSE = 0.67, *p* < 0.001, ${\text{η}}_{p}^{2}$ = 0.64, and a significant main effect of resolution, *F*(4,76) = 36.79, MSE = 0.32, *p* < 0.001, ${\text{η}}_{p}^{2}$ = 0.66. There was also a significant interaction between these two factors, *F*(4,76) = 3.05, MSE = 0.26, *p* = 0.02, ${\text{η}}_{p}^{2}$ = 0.14. Investigation of this interaction, using separate ANOVAs for each of the two memory load levels, revealed that the mean difference (MD) between R1 and R5 was statistically significant at both load levels, high: MD = 1.49, *p* < 0.001; low: MD = 0.92, *p* < 0.001. However, between R1 and R4, there was a statistically significant difference at high load: MD = 0.63, *p* = 0.007 but not at low load: MD = 0.15, *p* = 0.28, see Figure 2. It is also interesting to note that performance at R5 differed significantly from performance at all other levels of resolution at both memory loads, all *p*s < 0.002. Further, performance at R4 differed significantly from performance at R3 for both memory loads, high: MD = 0.43, *p* = 0.03; low: MD = 0.29, *p* = 0.04. However, although there was a tendency for performance at R3 to be lower than at R2 when working memory load was high, MD = 0.34, *p* = 0.07; there was no difference when load was low, MD = 0.06, *p* = 0.52. This pattern demonstrates that working memory for manual gestures is more sensitive to resolution when working memory load is high than when it is low.

Response bias was analyzed by calculating *c* (Stanislaw and Todorov, 1999). The grand mean *c*-value was 0.14 (SD = 0.07) which was significantly different from the neutral point (0), *t*(19) = 9.45, *p* < 0.001. Repeated measures ANOVA showed no main effect of *n*, *F*(1,19) = 1.69, MSE = 0.05, *p* = 0.21, a significant but small main effect of resolution, *F*(4,76) = 3.55, MSE = 0.04, *p* = 0.01, ${\text{η}}_{p}^{2}$ = 0.16, and, importantly, no significant interaction, *F*(4,76) = 0.88, MSE = 0.04, *p* = 0.48. Pairwise comparisons showed that response bias at R1, *c* = 0.20, was significantly greater than at R5 (*c* = 0.07), *p* = 0.01, demonstrating an increasing bias toward a positive response as resolution decreased.

### Computer Games

**FIGURE 3:**
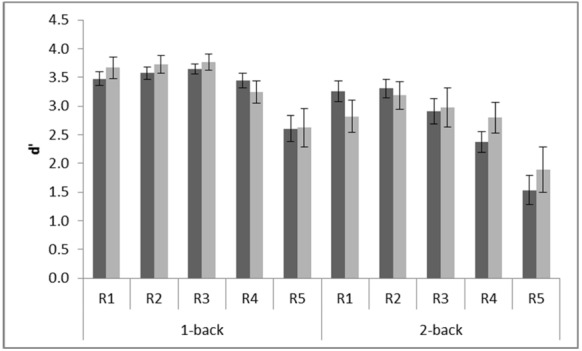
**Interaction between experience of playing computer games and performance on the *n*-back working memory task.** R1 (720 × 480 pixels); R2 (180 × 120 pixels); R3 (90 × 60 pixels); R4 (24 × 16 pixels); R5 (12 × 8 pixels). Dark bars show mean performance for participants who did not play computer games (*n* = 14) and light bars for those who did (*n* = 6). Error bars show standard error.

Playing action video games improves performance in a range of attentional, perceptual and cognitive tasks (Bejjanki et al., 2014). Therefore, we investigated whether experience playing computer games improved performance on the *n*-back task. Only six out of the 20 participants reported that they played computer games. Among those six, two reported playing 2 h daily, one reported playing 1 h and the other three played half an hour each. To determine whether playing computer games was associated with *n*-back performance, a between group variable was entered into the ANOVA based on whether the participant reported playing computer games or not. There was no main effect of playing computer games, *F*(1,18) = 0.11 MSE = 1.27, *p* = 0.75. However, there was a tendency toward a three-way interaction with working memory load and resolution, *F*(4,72) = 2.14, MSE = 0.25, *p* = 0.09, ${\text{η}}_{p}^{2}$ = 0.11, see Figure 3. Visual inspection of the interaction suggests that playing computer games may improve performance when memory load is high. To investigate this, we tested MD in performance between R5 and each of the other resolution levels for each group at the high load level. This revealed that although performance at R5 differed significantly from performance at all other levels of resolution at high memory load for non-players, all *p*s < 0.02, there was no significant difference in performance between R5 and R4 for computer gamers, *p* = 0.31. This pattern suggests that computer gamers may be less sensitive to resolution when working memory load is high than non-players.

To ensure that experience of playing computer games was not confounded by other variables we performed two-tailed independent samples *t*-tests to test for differences between the sub group who played computer games and the sub-group who did not. We found no statistically significant differences in age, *t*(18) = 0.98, *p* = 0.34 or Block Design, *t*(18) = 0.17, *p* = 0.86. The two Asian students stated that they did not play computer games. Two of the computer gamers were women.

## Discussion

In the present study, sign-naïve participants performed an *n*-back working memory task based on videos of lexical signs. The distinctness of the stimuli and working memory load were manipulated orthogonally by varying the resolution of the videos and presenting 1-back and 2-back versions of the task in a balanced within-subjects design.

In line with our prediction, poor visual resolution and high load resulted in poorer *n*-back performance. Moreover, and also in line with our prediction, we demonstrated an over-additive interaction such that the effect of reducing visual resolution was greater when load was high. This indicates that the shared mechanism supporting processing of distinctness and load previously identified for speech processing (Obleser et al., 2012; Petersen et al., 2015) can be generalized across language modality to sign processing. What is more, it indicates that this mechanism is not dependent on pre-existing semantic representation.

Due to the scarcity of previous work on the effect of reducing visual resolution on working memory for manual gestures, our choice of resolution levels was arbitrary: R1 (720 × 480 = 34560 pixels); R2 (180 × 120 = 21600 pixels); R3 (90 × 60 = 5400 pixels); R4 (24 × 16 = 384 pixels); R5 (12 × 8 = 96 pixels). There was no difference in performance between R1, R2, and R3 at either load level. However, there was a statistically significant difference in performance between R1 and R5 when resolution (number of pixels) was reduced by more than 99% at both load levels and a statistically significant difference in performance between R1 and R4 when resolution (number of pixels) was reduced by just under 99% at high load but not at low load. Thus, a considerable reduction in resolution was required before working memory performance was affected at either memory load. This suggests that representations adequate to solve the task could be generated even at very low resolution. In the present study, we used stimuli that are lexicalized signs and the participants were non-signers. We used this approach because it has been shown that neurocognitive representation of lexical signs is different from that of non-signs, even in non-signers (Cardin et al., 2015), and that pre-existing semantic representation enhances working memory for manual gestures (Rudner et al., under review). It is likely that sign language users who have pre-existing representations of lexical signs will have more robust performance at lower resolutions than non-signers. Future work should use sign language to investigate the interaction between load, distinctness and pre-existing representation.

In a recent study (Rudner et al., under review), we showed that deafness mitigates the effect of increasing working memory load manipulated using an *n*-back task based on manual gestures. In that study, n was manipulated at three levels (*n* = 1, 2, 3). We found that although signers were able to perform above chance when load was high at *n* = 3, the performance of non-signers was significantly lower. Thus, in the present study, we decided to use only two load levels, *n* = 1 and *n* = 2, omitting *n* = 3, because we considered that the performance of the non-signers in the present study would be too poor to reveal any further effects of stimulus degradation. However, Obleser et al. (2012) showed a potentiation of alpha power when working memory load was high and distinctness was low. Using a similar paradigm, Petersen et al. (2015) showed that hearing loss also increased alpha power, but that when load was high and distinctness was low, alpha power actually dropped for the individuals with the most severe degree of hearing loss, despite amplification. This was interpreted as indicating a breakdown in the mechanism supporting working memory at high load when stimulus distinctness is poor. Further, language modality-specific differences in working memory processing have been shown to emerge when cognitive demands are high (Rudner and Rönnberg, 2008). Thus, future studies should investigate how differing degrees of sensory acuity and long-term sensory deprivation with and without technical intervention interact with load, distinctness and pre-existing representation.

There has been considerable interest in cognitive training and its potential for increasing the performance in various domains of groups of individuals with functional impairments. Some studies have shown significant effects of cognitive training (e.g., Dahlin et al., 2008) but generally, transfer to other cognitive functions has been lacking (Owen et al., 2010). However, a body of work is now emerging that shows effects on cognition on videogaming (for an overview, see Bavelier and Davidson, 2013). In the present study, we asked the participants to report how many hours a day they spent playing computer games. We were surprised to find that only six out of the 20 participants played computer games at all. Notwithstanding, we found evidence to suggest that the individuals who stated that they played computer games were less affected by increasing levels of stimulus degradation when working memory load was high. Comparison of the two subgroups gives no grounds to suppose that these results are biased by age, gender, non-verbal intelligence or cultural background. This finding is in line with recent work showing superior attentional and oculomotor control generalizing to biologically relevant stimuli in students reporting playing action video games a minimum of three hours per week during the previous six months compared to matched non-players (Chisholm and Kingstone, 2015).

Bejjanki et al. (2014) suggested that videogaming may drive a general learning mechanism based on enhancement of perceptual templates. Such a mechanism might allow videogamers to establish better representations of degraded stimuli during a cognitive task. Further work should establish whether this mechanism does indeed allow non-signers to resist the negative effects of increasing load during working memory for manual gestures and whether such a mechanism is distinct from the mechanism that allows signers, with pre-existing lexical representations, to outperform non-signers on working memory for manual gestures (Rudner et al., under review). Future studies should investigate how the effect of videogaming on working memory for degraded manual gestures interacts with the effects of sign language experience.

## Conclusion

The results of the present study demonstrate that the over-additive interaction of load and distinctness predicted by the ELU model (Rönnberg et al., 2013) and empirically demonstrated for speech processing (Obleser et al., 2012; Petersen et al., 2015) can be generalized to sign processing. Moreover, we have shown that this interaction is not dependent on pre-existing semantic representation. Further, there was some evidence that the over-additive interaction was modulated by experience of playing computer games. This set of findings supports the notion of a shared working memory mechanism supporting load and distinctness and indicates that the mechanism is amodal. Future work using sign language should to investigate how the shared mechanism is modulated by pre-existing semantic representation as well as sensory factors and computer gaming experience.

### Conflict of Interest Statement

The authors declare that the research was conducted in the absence of any commercial or financial relationships that could be construed as a potential conflict of interest.
